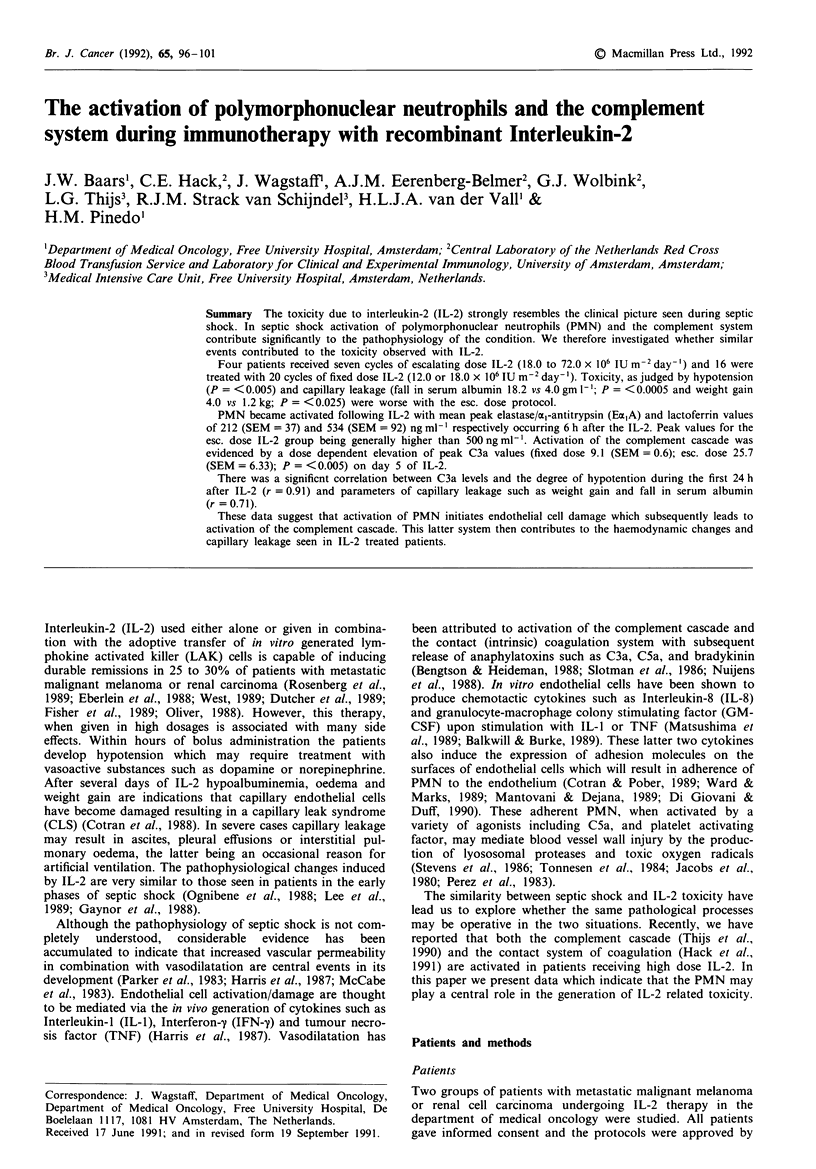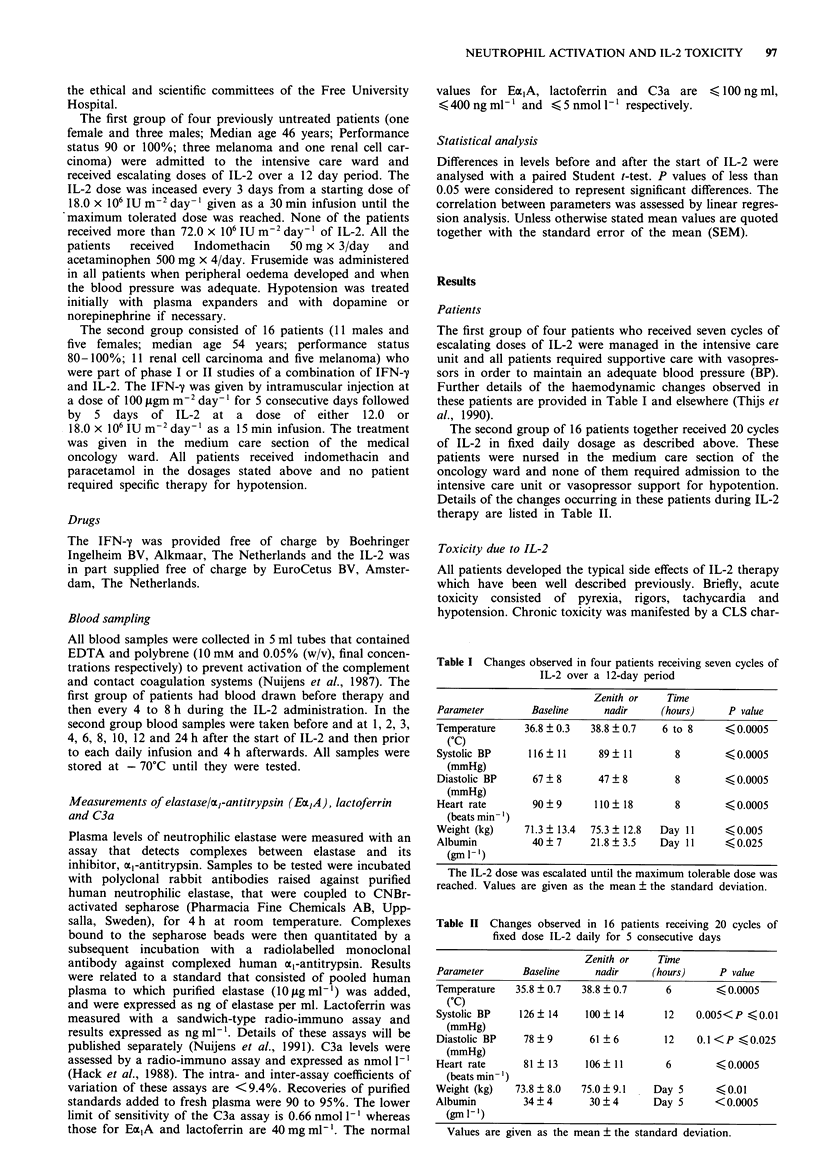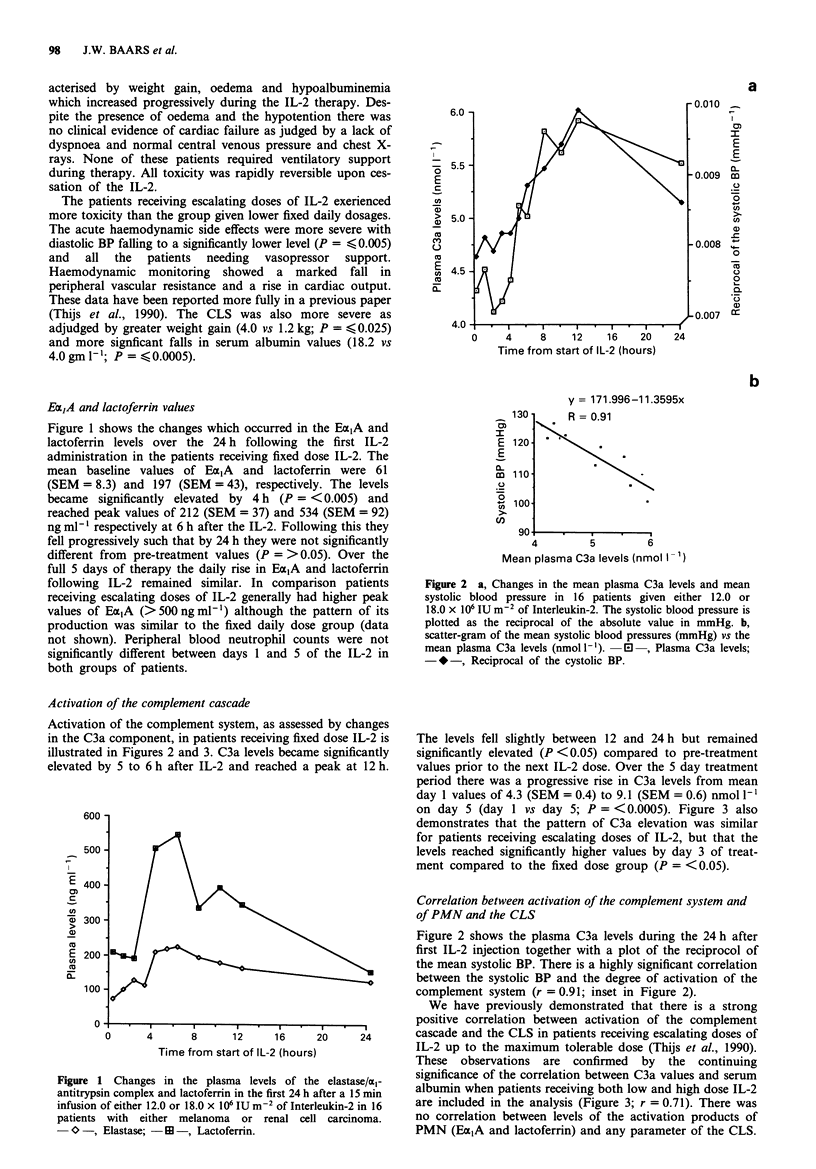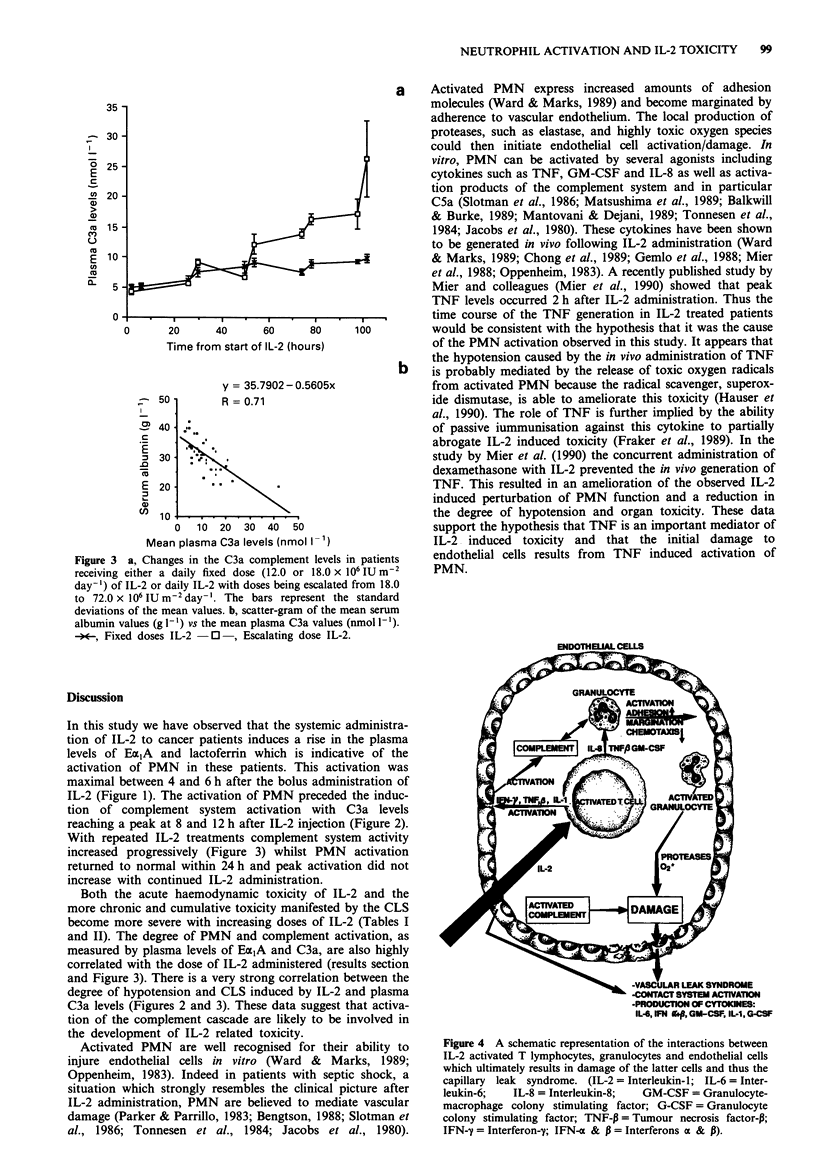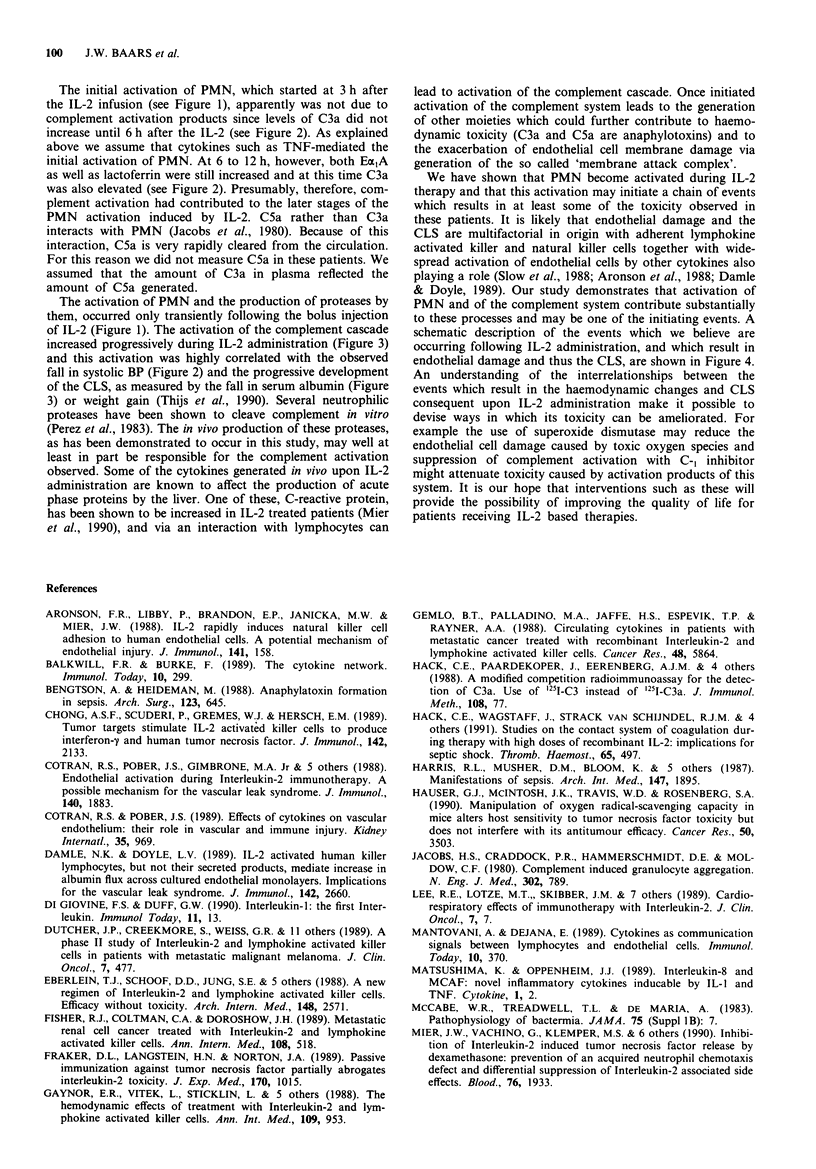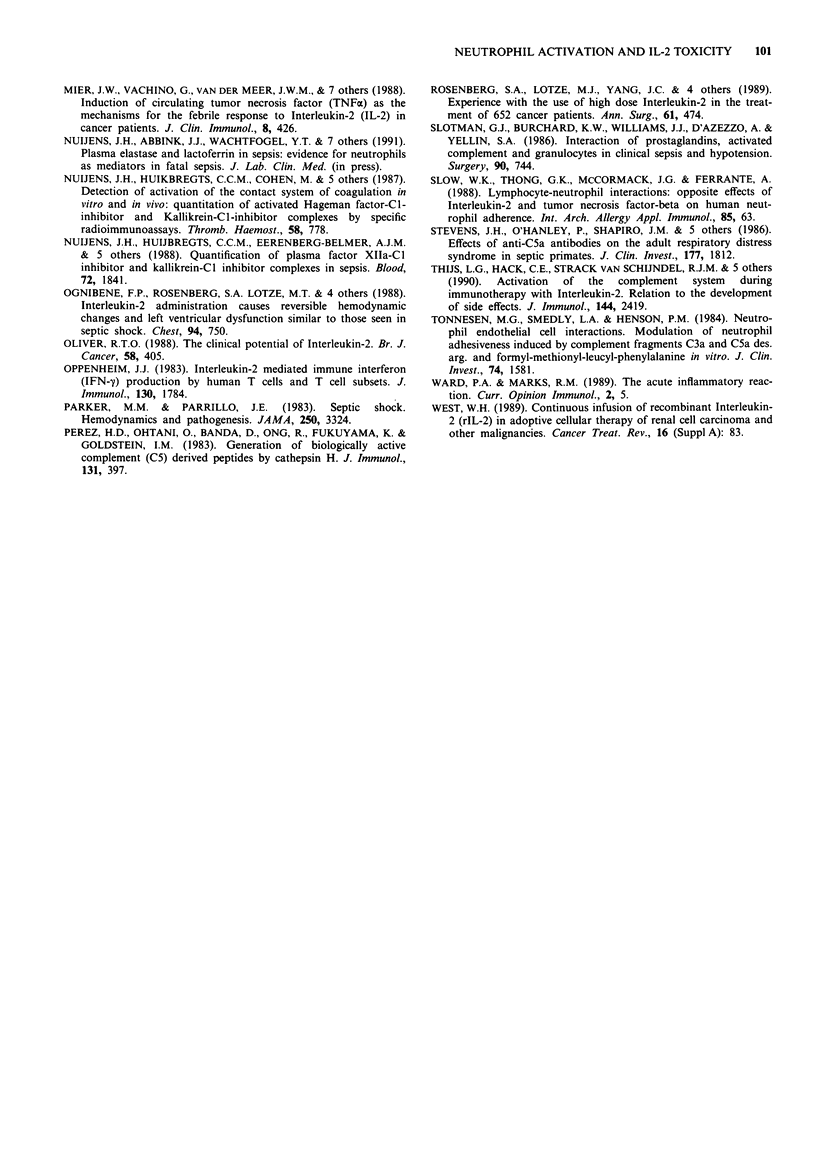# The activation of polymorphonuclear neutrophils and the complement system during immunotherapy with recombinant interleukin-2.

**DOI:** 10.1038/bjc.1992.18

**Published:** 1992-01

**Authors:** J. W. Baars, C. E. Hack, J. Wagstaff, A. J. Eerenberg-Belmer, G. J. Wolbink, L. G. Thijs, R. J. Strack van Schijndel, H. L. van der Vall, H. M. Pinedo

**Affiliations:** Department of Medical Oncology, Free University Hospital, Amsterdam, Netherlands.

## Abstract

The toxicity due to interleukin-2 (IL-2) strongly resembles the clinical picture seen during septic shock. In septic shock activation of polymorphonuclear neutrophils (PMN) and the complement system contribute significantly to the pathophysiology of the condition. We therefore investigated whether similar events contributed to the toxicity observed with IL-2. Four patients received seven cycles of escalating dose IL-2 (18.0 to 72.0 X 10(6) IU m-2 day-1) and 16 were treated with 20 cycles of fixed dose IL-2 (12.0 or 18.0 X 10(6) IU m-2 day-1). Toxicity, as judged by hypotension (P = less than 0.005) and capillary leakage (fall in serum albumin 18.2 vs 4.0 gm l-1; P = less than 0.0005 and weight gain 4.0 vs 1.2 kg; P = less than 0.025) were worse with the esc. dose protocol. PMN became activated following IL-2 with mean peak elastase/alpha 1-antitrypsin (E alpha 1 A) and lactoferrin values of 212 (SEM = 37) and 534 (SEM = 92) ng ml-1 respectively occurring 6 h after the IL-2. Peak values for the esc. dose IL-2 group being generally higher than 500 ng ml-1. Activation of the complement cascade was evidenced by a dose dependent elevation of peak C3a values (fixed dose 9.1 (SEM = 0.6); esc. dose 25.7 (SEM = 6.33); P = less than 0.005) on day 5 of IL-2. There was a significant correlation between C3a levels and the degree of hypotention during the first 24 h after IL-2 (r = 0.91) and parameters of capillary leakage such as weight gain and fall in serum albumin (r = 0.71). These data suggest that activation of PMN initiates endothelial cell damage which subsequently leads to activation of the complement cascade. This latter system then contributes to the haemodynamic changes and capillary leakage seen in IL-2 treated patients.


*Br. J. Cancer* (1992), **65**, 96–101 © Macmillan Press Ltd., 1992

### The activation of polymorphonuclear neutrophils and the complement system during immunotherapy with recombinant Interleukin-2

J.W. Baars[1], C.E. Hack,[2], J. Wagstaff[1], A.J.M. Eerenberg-Belmer[2], G.J. Wolbink[2], L.G. Thijs[3], R.J.M. Strack van Schijndel[3], H.L.J.A. van der Vall[1] & H.M. Pinedo[1]

[1]*Department of Medical Oncology, Free University Hospital, Amsterdam;* [2]*Central Laboratory of the Netherlands Red Cross Blood Transfusion Service and Laboratory for Clinical and Experimental Immunology, University of Amsterdam, Amsterdam;* [3]*Medical Intensive Care Unit, Free University Hospital, Amsterdam, Netherlands.*

**Summary** The toxicity due to interleukin-2 (IL-2) strongly resembles the clinical picture seen during septic shock. In septic shock activation of polymorphonuclear neutrophils (PMN) and the complement system contribute significantly to the pathophysiology of the condition. We therefore investigated whether similar events contributed to the toxicity observed with IL-2.

Four patients received seven cycles of escalating dose IL-2 (18.0 to $72.0 \times 10^6$ IU $m^{-2}$ $day^{-1}$) and 16 were treated with 20 cycles of fixed dose IL-2 (12.0 or $18.0 \times 10^6$ IU $m^{-2}$ $day^{-1}$). Toxicity, as judged by hypotension ($P = <0.005$) and capillary leakage (fall in serum albumin 18.2 *vs* 4.0 gm $l^{-1}$; $P = <0.0005$ and weight gain 4.0 *vs* 1.2 kg; $P = <0.025$) were worse with the esc. dose protocol.

PMN became activated following IL-2 with mean peak elastase/$\alpha_1$-antitrypsin (E$\alpha_1$A) and lactoferrin values of 212 (SEM = 37) and 534 (SEM = 92) ng $ml^{-1}$ respectively occurring 6 h after the IL-2. Peak values for the esc. dose IL-2 group being generally higher than 500 ng $ml^{-1}$. Activation of the complement cascade was evidenced by a dose dependent elevation of peak C3a values (fixed dose 9.1 (SEM = 0.6); esc. dose 25.7 (SEM = 6.33); $P = <0.005$) on day 5 of IL-2.

There was a significnt correlation between C3a levels and the degree of hypotention during the first 24 h after IL-2 ($r = 0.91$) and parameters of capillary leakage such as weight gain and fall in serum albumin ($r = 0.71$).

These data suggest that activation of PMN initiates endothelial cell damage which subsequently leads to activation of the complement cascade. This latter system then contributes to the haemodynamic changes and capillary leakage seen in IL-2 treated patients.

Interleukin-2 (IL-2) used either alone or given in combination with the adoptive transfer of *in vitro* generated lymphokine activated killer (LAK) cells is capable of inducing durable remissions in 25 to 30% of patients with metastatic malignant melanoma or renal carcinoma (Rosenberg *et al.*, 1989; Eberlein *et al.*, 1988; West, 1989; Dutcher *et al.*, 1989; Fisher *et al.*, 1989; Oliver, 1988). However, this therapy, when given in high dosages is associated with many side effects. Within hours of bolus administration the patients develop hypotension which may require treatment with vasoactive substances such as dopamine or norepinephrine. After several days of IL-2 hypoalbuminemia, oedema and weight gain are indications that capillary endothelial cells have become damaged resulting in a capillary leak syndrome (CLS) (Cotran *et al.*, 1988). In severe cases capillary leakage may result in ascites, pleural effusions or interstitial pulmonary oedema, the latter being an occasional reason for artificial ventilation. The pathophysiological changes induced by IL-2 are very similar to those seen in patients in the early phases of septic shock (Ognibene *et al.*, 1988; Lee *et al.*, 1989; Gaynor *et al.*, 1988).

Although the pathophysiology of septic shock is not completely understood, considerable evidence has been accumulated to indicate that increased vascular permeability in combination with vasodilatation are central events in its development (Parker *et al.*, 1983; Harris *et al.*, 1987; McCabe *et al.*, 1983). Endothelial cell activation/damage are thought to be mediated via the *in vivo* generation of cytokines such as Interleukin-1 (IL-1), Interferon-$\gamma$ (IFN-$\gamma$) and tumour necrosis factor (TNF) (Harris *et al.*, 1987). Vasodilatation has been attributed to activation of the complement cascade and the contact (intrinsic) coagulation system with subsequent release of anaphylatoxins such as C3a, C5a, and bradykinin (Bengtson & Heideman, 1988; Slotman *et al.*, 1986; Nuijens *et al.*, 1988). *In vitro* endothelial cells have been shown to produce chemotactic cytokines such as Interleukin-8 (IL-8) and granulocyte-macrophage colony stimulating factor (GM-CSF) upon stimulation with IL-1 or TNF (Matsushima *et al.*, 1989; Balkwill & Burke, 1989). These latter two cytokines also induce the expression of adhesion molecules on the surfaces of endothelial cells which will result in adherence of PMN to the endothelium (Cotran & Pober, 1989; Ward & Marks, 1989; Mantovani & Dejana, 1989; Di Giovani & Duff, 1990). These adherent PMN, when activated by a variety of agonists including C5a, and platelet activating factor, may mediate blood vessel wall injury by the production of lyososomal proteases and toxic oxygen radicals (Stevens *et al.*, 1986; Tonnesen *et al.*, 1984; Jacobs *et al.*, 1980; Perez *et al.*, 1983).

The similarity between septic shock and IL-2 toxicity have lead us to explore whether the same pathological processes may be operative in the two situations. Recently, we have reported that both the complement cascade (Thijs *et al.*, 1990) and the contact system of coagulation (Hack *et al.*, 1991) are activated in patients receiving high dose IL-2. In this paper we present data which indicate that the PMN may play a central role in the generation of IL-2 related toxicity.

### Patients and methods

### *Patients*

Two groups of patients with metastatic malignant melanoma or renal cell carcinoma undergoing IL-2 therapy in the department of medical oncology were studied. All patients gave informed consent and the protocols were approved by

Correspondence: J. Wagstaff, Department of Medical Oncology, Department of Medical Oncology, Free University Hospital, De Boelelaan 1117, 1081 HV Amsterdam, The Netherlands.
Received 17 June 1991; and in revised form 19 September 1991.

the ethical and scientific committees of the Free University Hospital.

The first group of four previously untreated patients (one female and three males; Median age 46 years; Performance status 90 or 100%; three melanoma and one renal cell carcinoma) were admitted to the intensive care ward and received escalating doses of IL-2 over a 12 day period. The IL-2 dose was inceased every 3 days from a starting dose of $18.0 \times 10^6$ IU $m^{-2}$ $day^{-1}$ given as a 30 min infusion until the maximum tolerated dose was reached. None of the patients received more than $72.0 \times 10^6$ IU $m^{-2}$ $day^{-1}$ of IL-2. All the patients received Indomethacin 50 mg × 3/day and acetaminophen 500 mg × 4/day. Frusemide was administered in all patients when peripheral oedema developed and when the blood pressure was adequate. Hypotension was treated initially with plasma expanders and with dopamine or norepinephrine if necessary.

The second group consisted of 16 patients (11 males and five females; median age 54 years; performance status 80-100%; 11 renal cell carcinoma and five melanoma) who were part of phase I or II studies of a combination of IFN-γ and IL-2. The IFN-γ was given by intramuscular injection at a dose of 100 μgm $m^{-2}$ $day^{-1}$ for 5 consecutive days followed by 5 days of IL-2 at a dose of either 12.0 or $18.0 \times 10^6$ IU $m^{-2}$ $day^{-1}$ as a 15 min infusion. The treatment was given in the medium care section of the medical oncology ward. All patients received indomethacin and paracetamol in the dosages stated above and no patient required specific therapy for hypotension.

### *Drugs*

The IFN-γ was provided free of charge by Boehringer Ingelheim BV, Alkmaar, The Netherlands and the IL-2 was in part supplied free of charge by EuroCetus BV, Amsterdam, The Netherlands.

### *Blood sampling*

All blood samples were collected in 5 ml tubes that contained EDTA and polybrene (10 mM and 0.05% (w/v), final concentrations respectively) to prevent activation of the complement and contact coagulation systems (Nuijens *et al.*, 1987). The first group of patients had blood drawn before therapy and then every 4 to 8 h during the IL-2 administration. In the second group blood samples were taken before and at 1, 2, 3, 4, 6, 8, 10, 12 and 24 h after the start of IL-2 and then prior to each daily infusion and 4 h afterwards. All samples were stored at − 70°C until they were tested.

### *Measurements of elastase/α₁-antitrypsin (Eα₁A), lactoferrin and C3a*

Plasma levels of neutrophilic elastase were measured with an assay that detects complexes between elastase and its inhibitor, $\alpha_1$-antitrypsin. Samples to be tested were incubated with polyclonal rabbit antibodies raised against purified human neutrophilic elastase, that were coupled to CNBr-activated sepharose (Pharmacia Fine Chemicals AB, Uppsalla, Sweden), for 4 h at room temperature. Complexes bound to the sepharose beads were then quantitated by a subsequent incubation with a radiolabelled monoclonal antibody against complexed human $\alpha_1$-antitrypsin. Results were related to a standard that consisted of pooled human plasma to which purified elastase (10 μg $ml^{-1}$) was added, and were expressed as ng of elastase per ml. Lactoferrin was measured with a sandwich-type radio-immuno assay and results expressed as ng $ml^{-1}$. Details of these assays will be published separately (Nuijens *et al.*, 1991). C3a levels were assessed by a radio-immuno assay and expressed as nmol $l^{-1}$ (Hack *et al.*, 1988). The intra- and inter-assay coefficients of variation of these assays are <9.4%. Recoveries of purified standards added to fresh plasma were 90 to 95%. The lower limit of sensitivity of the C3a assay is 0.66 nmol $l^{-1}$ whereas those for $E\alpha_1A$ and lactoferrin are 40 mg $ml^{-1}$. The normal values for $E\alpha_1A$, lactoferrin and C3a are ⩽100 ng ml, ⩽400 ng $ml^{-1}$ and ⩽5 nmol $l^{-1}$ respectively.

### *Statistical analysis*

Differences in levels before and after the start of IL-2 were analysed with a paired Student *t*-test. *P* values of less than 0.05 were considered to represent significant differences. The correlation between parameters was assessed by linear regression analysis. Unless otherwise stated mean values are quoted together with the standard error of the mean (SEM).

### Results

### *Patients*

The first group of four patients who received seven cycles of escalating doses of IL-2 were managed in the intensive care unit and all patients required supportive care with vasopressors in order to maintain an adequate blood pressure (BP). Further details of the haemodynamic changes observed in these patients are provided in Table I and elsewhere (Thijs *et al.*, 1990).

The second group of 16 patients together received 20 cycles of IL-2 in fixed daily dosage as described above. These patients were nursed in the medium care section of the oncology ward and none of them required admission to the intensive care unit or vasopressor support for hypotention. Details of the changes occurring in these patients during IL-2 therapy are listed in Table II.

### *Toxicity due to IL-2*

All patients developed the typical side effects of IL-2 therapy which have been well described previously. Briefly, acute toxicity consisted of pyrexia, rigors, tachycardia and hypotension. Chronic toxicity was manifested by a CLS char-

**Table I** Changes observed in four patients receiving seven cycles of IL-2 over a 12-day period

| *Parameter* | *Baseline* | *Zenith or nadir* | *Time (hours)* | *P value* |
|---|---|---|---|---|
| Temperature (°C) | 36.8 ± 0.3 | 38.8 ± 0.7 | 6 to 8 | ⩽0.0005 |
| Systolic BP (mmHg) | 116 ± 11 | 89 ± 11 | 8 | ⩽0.0005 |
| Diastolic BP (mmHg) | 67 ± 8 | 47 ± 8 | 8 | ⩽0.0005 |
| Heart rate (beats $min^{-1}$) | 90 ± 9 | 110 ± 18 | 8 | ⩽0.0005 |
| Weight (kg) | 71.3 ± 13.4 | 75.3 ± 12.8 | Day 11 | ⩽0.005 |
| Albumin (gm $l^{-1}$) | 40 ± 7 | 21.8 ± 3.5 | Day 11 | ⩽0.025 |

The IL-2 dose was escalated until the maximum tolerable dose was reached. Values are given as the mean ± the standard deviation.

**Table II** Changes observed in 16 patients receiving 20 cycles of fixed dose IL-2 daily for 5 consecutive days

| *Parameter* | *Baseline* | *Zenith or nadir* | *Time (hours)* | *P value* |
|---|---|---|---|---|
| Temperature (°C) | 35.8 ± 0.7 | 38.8 ± 0.7 | 6 | ⩽0.0005 |
| Systolic BP (mmHg) | 126 ± 14 | 100 ± 14 | 12 | 0.005<*P* ⩽0.01 |
| Diastolic BP (mmHg) | 78 ± 9 | 61 ± 6 | 12 | 0.1<*P* ⩽0.025 |
| Heart rate (beats $min^{-1}$) | 81 ± 13 | 106 ± 11 | 6 | ⩽0.0005 |
| Weight (kg) | 73.8 ± 8.0 | 75.0 ± 9.1 | Day 5 | ⩽0.01 |
| Albumin (gm $l^{-1}$) | 34 ± 4 | 30 ± 4 | Day 5 | <0.0005 |

Values are given as the mean ± the standard deviation.

acterised by weight gain, oedema and hypoalbuminemia which increased progressively during the IL-2 therapy. Despite the presence of oedema and the hypotention there was no clinical evidence of cardiac failure as judged by a lack of dyspnoea and normal central venous pressure and chest X-rays. None of these patients required ventilatory support during therapy. All toxicity was rapidly reversible upon cessation of the IL-2.

The patients receiving escalating doses of IL-2 exerienced more toxicity than the group given lower fixed daily dosages. The acute haemodynamic side effects were more severe with diastolic BP falling to a significantly lower level ($P = \leqslant 0.005$) and all the patients needing vasopressor support. Haemodynamic monitoring showed a marked fall in peripheral vascular resistance and a rise in cardiac output. These data have been reported more fully in a previous paper (Thijs *et al.*, 1990). The CLS was also more severe as adjudged by greater weight gain (4.0 *vs* 1.2 kg; $P = \leqslant 0.025$) and more signficant falls in serum albumin values (18.2 *vs* 4.0 gm $l^{-1}$; $P = \leqslant 0.0005$).

### *E$\alpha_1$A and lactoferrin values*

Figure 1 shows the changes which occurred in the E$\alpha_1$A and lactoferrin levels over the 24 h following the first IL-2 administration in the patients receiving fixed dose IL-2. The mean baseline values of E$\alpha_1$A and lactoferrin were 61 (SEM = 8.3) and 197 (SEM = 43), respectively. The levels became significantly elevated by 4 h ($P = < 0.005$) and reached peak values of 212 (SEM = 37) and 534 (SEM = 92) ng $ml^{-1}$ respectively at 6 h after the IL-2. Following this they fell progressively such that by 24 h they were not significantly different from pre-treatment values ($P = > 0.05$). Over the full 5 days of therapy the daily rise in E$\alpha_1$A and lactoferrin following IL-2 remained similar. In comparison patients receiving escalating doses of IL-2 generally had higher peak values of E$\alpha_1$A (> 500 ng $ml^{-1}$) although the pattern of its production was similar to the fixed daily dose group (data not shown). Peripheral blood neutrophil counts were not significantly different between days 1 and 5 of the IL-2 in both groups of patients.

### *Activation of the complement cascade*

Activation of the complement system, as assessed by changes in the C3a component, in patients receiving fixed dose IL-2 is illustrated in Figures 2 and 3. C3a levels became significantly elevated by 5 to 6 h after IL-2 and reached a peak at 12 h.

**Figure 1** Changes in the plasma levels of the elastase/$\alpha_1$-antitrypsin complex and lactoferrin in the first 24 h after a 15 min infusion of either 12.0 or 18.0 × $10^6$ IU $m^{-2}$ of Interleukin-2 in 16 patients with either melanoma or renal cell carcinoma. —◇—, Elastase; —⊞—, Lactoferrin.

**Figure 2** **a**, Changes in the mean plasma C3a levels and mean systolic blood pressure in 16 patients given either 12.0 or 18.0 × $10^6$ IU $m^{-2}$ of Interleukin-2. The systolic blood pressure is plotted as the reciprocal of the absolute value in mmHg. **b**, scatter-gram of the mean systolic blood pressures (mmHg) *vs* the mean plasma C3a levels (nmol $l^{-1}$). —⊡—, Plasma C3a levels; —◆—, Reciprocal of the cystolic BP.

The levels fell slightly between 12 and 24 h but remained significantly elevated ($P < 0.05$) compared to pre-treatment values prior to the next IL-2 dose. Over the 5 day treatment period there was a progressive rise in C3a levels from mean day 1 values of 4.3 (SEM = 0.4) to 9.1 (SEM = 0.6) nmol $l^{-1}$ on day 5 (day 1 *vs* day 5; $P = < 0.0005$). Figure 3 also demonstrates that the pattern of C3a elevation was similar for patients receiving escalating doses of IL-2, but that the levels reached significantly higher values by day 3 of treatment compared to the fixed dose group ($P = < 0.05$).

### *Correlation between activation of the complement system and of PMN and the CLS*

Figure 2 shows the plasma C3a levels during the 24 h after first IL-2 injection together with a plot of the reciprocol of the mean systolic BP. There is a highly significant correlation between the systolic BP and the degree of activation of the complement system ($r = 0.91$; inset in Figure 2).

We have previously demonstrated that there is a strong positive correlation between activation of the complement cascade and the CLS in patients receiving escalating doses of IL-2 up to the maximum tolerable dose (Thijs *et al.*, 1990). These observations are confirmed by the continuing significance of the correlation between C3a values and serum albumin when patients receiving both low and high dose IL-2 are included in the analysis (Figure 3; $r = 0.71$). There was no correlation between levels of the activation products of PMN (E$\alpha_1$A and lactoferrin) and any parameter of the CLS.

**Figure 3** **a**, Changes in the C3a complement levels in patients receiving either a daily fixed dose (12.0 or $18.0 \times 10^6$ IU $m^{-2}$ $day^{-1}$) of IL-2 or daily IL-2 with doses being escalated from 18.0 to $72.0 \times 10^6$ IU $m^{-2}$ $day^{-1}$. The bars represent the standard deviations of the mean values. **b**, scatter-gram of the mean serum albumin values (g $l^{-1}$) *vs* the mean plasma C3a values (nmol $l^{-1}$). —×—, Fixed doses IL-2 —□—, Escalating dose IL-2.

### Discussion

In this study we have observed that the systemic administration of IL-2 to cancer patients induces a rise in the plasma levels of $E\alpha_1A$ and lactoferrin which is indicative of the activation of PMN in these patients. This activation was maximal between 4 and 6 h after the bolus administration of IL-2 (Figure 1). The activation of PMN preceded the induction of complement system activation with C3a levels reaching a peak at 8 and 12 h after IL-2 injection (Figure 2). With repeated IL-2 treatments complement system activity increased progressively (Figure 3) whilst PMN activation returned to normal within 24 h and peak activation did not increase with continued IL-2 administration.

Both the acute haemodynamic toxicity of IL-2 and the more chronic and cumulative toxicity manifested by the CLS become more severe with increasing doses of IL-2 (Tables I and II). The degree of PMN and complement activation, as measured by plasma levels of $E\alpha_1A$ and C3a, are also highly correlated with the dose of IL-2 administered (results section and Figure 3). There is a very strong correlation between the degree of hypotension and CLS induced by IL-2 and plasma C3a levels (Figures 2 and 3). These data suggest that activation of the complement cascade are likely to be involved in the development of IL-2 related toxicity.

Activated PMN are well recognised for their ability to injure endothelial cells *in vitro* (Ward & Marks, 1989; Oppenheim, 1983). Indeed in patients with septic shock, a situation which strongly resembles the clinical picture after IL-2 administration, PMN are believed to mediate vascular damage (Parker & Parrillo, 1983; Bengtson, 1988; Slotman *et al.*, 1986; Tonnesen *et al.*, 1984; Jacobs *et al.*, 1980). Activated PMN express increased amounts of adhesion molecules (Ward & Marks, 1989) and become marginated by adherence to vascular endothelium. The local production of proteases, such as elastase, and highly toxic oxygen species could then initiate endothelial cell activation/damage. *In vitro*, PMN can be activated by several agonists including cytokines such as TNF, GM-CSF and IL-8 as well as activation products of the complement system and in particular C5a (Slotman *et al.*, 1986; Matsushima *et al.*, 1989; Balkwill & Burke, 1989; Mantovani & Dejani, 1989; Tonnesen *et al.*, 1984; Jacobs *et al.*, 1980). These cytokines have been shown to be generated *in vivo* following IL-2 administration (Ward & Marks, 1989; Chong *et al.*, 1989; Gemlo *et al.*, 1988; Mier *et al.*, 1988; Oppenheim, 1983). A recently published study by Mier and colleagues (Mier *et al.*, 1990) showed that peak TNF levels occurred 2 h after IL-2 administration. Thus the time course of the TNF generation in IL-2 treated patients would be consistent with the hypothesis that it was the cause of the PMN activation observed in this study. It appears that the hypotension caused by the *in vivo* administration of TNF is probably mediated by the release of toxic oxygen radicals from activated PMN because the radical scavenger, superoxide dismutase, is able to ameliorate this toxicity (Hauser *et al.*, 1990). The role of TNF is further implied by the ability of passive iummunisation against this cytokine to partially abrogate IL-2 induced toxicity (Fraker *et al.*, 1989). In the study by Mier *et al.* (1990) the concurrent administration of dexamethasone with IL-2 prevented the *in vivo* generation of TNF. This resulted in an amelioration of the observed IL-2 induced perturbation of PMN function and a reduction in the degree of hypotension and organ toxicity. These data support the hypothesis that TNF is an important mediator of IL-2 induced toxicity and that the initial damage to endothelial cells results from TNF induced activation of PMN.

**Figure 4** A schematic representation of the interactions between IL-2 activated T lymphocytes, granulocytes and endothelial cells which ultimately results in damage of the latter cells and thus the capillary leak syndrome. (IL-2 = Interleukin-1; IL-6 = Interleukin-6; IL-8 = Interleukin-8; GM-CSF = Granulocyte-macrophage colony stimulating factor; G-CSF = Granulocyte colony stimulating factor; TNF-β = Tumour necrosis factor-β; IFN-γ = Interferon-γ; IFN-α & β = Interferons α & β).

The initial activation of PMN, which started at 3 h after the IL-2 infusion (see Figure 1), apparently was not due to complement activation products since levels of C3a did not increase until 6 h after the IL-2 (see Figure 2). As explained above we assume that cytokines such as TNF-mediated the initial activation of PMN. At 6 to 12 h, however, both E$\alpha_1$A as well as lactoferrin were still increased and at this time C3a was also elevated (see Figure 2). Presumably, therefore, complement activation had contributed to the later stages of the PMN activation induced by IL-2. C5a rather than C3a interacts with PMN (Jacobs *et al.*, 1980). Because of this interaction, C5a is very rapidly cleared from the circulation. For this reason we did not measure C5a in these patients. We assumed that the amount of C3a in plasma reflected the amount of C5a generated.

The activation of PMN and the production of proteases by them, occurred only transiently following the bolus injection of IL-2 (Figure 1). The activation of the complement cascade increased progressively during IL-2 administration (Figure 3) and this activation was highly correlated with the observed fall in systolic BP (Figure 2) and the progressive development of the CLS, as measured by the fall in serum albumin (Figure 3) or weight gain (Thijs *et al.*, 1990). Several neutrophilic proteases have been shown to cleave complement *in vitro* (Perez *et al.*, 1983). The *in vivo* production of these proteases, as has been demonstrated to occur in this study, may well at least in part be responsible for the complement activation observed. Some of the cytokines generated *in vivo* upon IL-2 administration are known to affect the production of acute phase proteins by the liver. One of these, C-reactive protein, has been shown to be increased in IL-2 treated patients (Mier *et al.*, 1990), and via an interaction with lymphocytes can lead to activation of the complement cascade. Once initiated activation of the complement system leads to the generation of other moieties which could further contribute to haemodynamic toxicity (C3a and C5a are anaphylotoxins) and to the exacerbation of endothelial cell membrane damage via generation of the so called 'membrane attack complex'.

We have shown that PMN become activated during IL-2 therapy and that this activation may initiate a chain of events which results in at least some of the toxicity observed in these patients. It is likely that endothelial damage and the CLS are multifactorial in origin with adherent lymphokine activated killer and natural killer cells together with widespread activation of endothelial cells by other cytokines also playing a role (Slow *et al.*, 1988; Aronson *et al.*, 1988; Damle & Doyle, 1989). Our study demonstrates that activation of PMN and of the complement system contribute substantially to these processes and may be one of the initiating events. A schematic description of the events which we believe are occurring following IL-2 administration, and which result in endothelial damage and thus the CLS, are shown in Figure 4. An understanding of the interrelationships between the events which result in the haemodynamic changes and CLS consequent upon IL-2 administration make it possible to devise ways in which its toxicity can be ameliorated. For example the use of superoxide dismutase may reduce the endothelial cell damage caused by toxic oxygen species and suppression of complement activation with C-$_1$ inhibitor might attenuate toxicity caused by activation products of this system. It is our hope that interventions such as these will provide the possibility of improving the quality of life for patients receiving IL-2 based therapies.

### References

ARONSON, F.R., LIBBY, P., BRANDON, E.P., JANICKA, M.W. & MIER, J.W. (1988). IL-2 rapidly induces natural killer cell adhesion to human endothelial cells. A potential mechanism of endothelial injury. *J. Immunol.*, **141**, 158.

BALKWILL, F.R. & BURKE, F. (1989). The cytokine network. *Immunol. Today*, **10**, 299.

BENGTSON, A. & HEIDEMAN, M. (1988). Anaphylatoxin formation in sepsis. *Arch. Surg.*, **123**, 645.

CHONG, A.S.F., SCUDERI, P., GREMES, W.J. & HERSCH, E.M. (1989). Tumor targets stimulate IL-2 activated killer cells to produce interferon-γ and human tumor necrosis factor. *J. Immunol.*, **142**, 2133.

COTRAN, R.S., POBER, J.S., GIMBRONE, M.A. Jr & 5 others (1988). Endothelial activation during Interleukin-2 immunotherapy. A possible mechanism for the vascular leak syndrome. *J. Immunol.*, **140**, 1883.

COTRAN, R.S. & POBER, J.S. (1989). Effects of cytokines on vascular endothelium: their role in vascular and immune injury. *Kidney Internatl.*, **35**, 969.

DAMLE, N.K. & DOYLE, L.V. (1989). IL-2 activated human killer lymphocytes, but not their secreted products, mediate increase in albumin flux across cultured endothelial monolayers. Implications for the vascular leak syndrome. *J. Immunol.*, **142**, 2660.

DI GIOVINE, F.S. & DUFF, G.W. (1990). Interleukin-1: the first Interleukin. *Immunol Today*, **11**, 13.

DUTCHER, J.P., CREEKMORE, S., WEISS, G.R. & 11 others (1989). A phase II study of Interleukin-2 and lymphokine activated killer cells in patients with metastatic malignant melanoma. *J. Clin. Oncol.*, **7**, 477.

EBERLEIN, T.J., SCHOOF, D.D., JUNG, S.E. & 5 others (1988). A new regimen of Interleukin-2 and lymphokine activated killer cells. Efficacy without toxicity. *Arch. Intern. Med.*, **148**, 2571.

FISHER, R.J., COLTMAN, C.A. & DOROSHOW, J.H. (1989). Metastatic renal cell cancer treated with Interleukin-2 and lymphokine activated killer cells. *Ann. Intern. Med.*, **108**, 518.

FRAKER, D.L., LANGSTEIN, H.N. & NORTON, J.A. (1989). Passive immunization against tumor necrosis factor partially abrogates interleukin-2 toxicity. *J. Exp. Med.*, **170**, 1015.

GAYNOR, E.R., VITEK, L., STICKLIN, L. & 5 others (1988). The hemodynamic effects of treatment with Interleukin-2 and lymphokine activated killer cells. *Ann. Int. Med.*, **109**, 953.

GEMLO, B.T., PALLADINO, M.A., JAFFE, H.S., ESPEVIK, T.P. & RAYNER, A.A. (1988). Circulating cytokines in patients with metastatic cancer treated with recombinant Interleukin-2 and lymphokine activated killer cells. *Cancer Res.*, **48**, 5864.

HACK, C.E., PAARDEKOPER, J., EERENBERG, A.J.M. & 4 others (1988). A modified competition radioimmunoassay for the detection of C3a. Use of $^{125}$I-C3 instead of $^{125}$I-C3a. *J. Immunol. Meth.*, **108**, 77.

HACK, C.E., WAGSTAFF, J., STRACK VAN SCHIJNDEL, R.J.M. & 4 others (1991). Studies on the contact system of coagulation during therapy with high doses of recombinant IL-2: implications for septic shock. *Thromb. Haemost.*, **65**, 497.

HARRIS, R.L., MUSHER, D.M., BLOOM, K. & 5 others (1987). Manifestations of sepsis. *Arch. Int. Med.*, **147**, 1895.

HAUSER, G.J., MCINTOSH, J.K., TRAVIS, W.D. & ROSENBERG, S.A. (1990). Manipulation of oxygen radical-scavenging capacity in mice alters host sensitivity to tumor necrosis factor toxicity but does not interfere with its antitumour efficacy. *Cancer Res.*, **50**, 3503.

JACOBS, H.S., CRADDOCK, P.R., HAMMERSCHMIDT, D.E. & MOLDOW, C.F. (1980). Complement induced granulocyte aggregation. *N. Eng. J. Med.*, **302**, 789.

LEE, R.E., LOTZE, M.T.,, SKIBBER, J.M. & 7 others (1989). Cardiorespiratory effects of immunotherapy with Interleukin-2. *J. Clin. Oncol.*, **7**, 7.

MANTOVANI, A. & DEJANA, E. (1989). Cytokines as communication signals between lymphocytes and endothelial cells. *Immunol. Today*, **10**, 370.

MATSUSHIMA, K. & OPPENHEIM, J.J. (1989). Interleukin-8 and MCAF: novel inflammatory cytokines inducable by IL-1 and TNF. *Cytokine*, **1**, 2.

McCABE, W.R., TREADWELL, T.L. & DE MARIA, A. (1983). Pathophysiology of bactermia. *JAMA*. **75** (Suppl 1B): 7.

MIER, J.W., VACHINO, G., KLEMPER, M.S. & 6 others (1990). Inhibition of Interleukin-2 induced tumor necrosis factor release by dexamethasone: prevention of an acquired neutrophil chemotaxis defect and differential suppression of Interleukin-2 associated side effects. *Blood.*, **76**, 1933.

MIER, J.W., VACHINO, G., VAN DER MEER, J.W.M., & 7 others (1988). Induction of circulating tumor necrosis factor (TNFα) as the mechanisms for the febrile response to Interleukin-2 (IL-2) in cancer patients. *J. Clin. Immunol.*, **8**, 426.

NUIJENS, J.H., ABBINK, J.J., WACHTFOGEL, Y.T. & 7 others (1991). Plasma elastase and lactoferrin in sepsis: evidence for neutrophils as mediators in fatal sepsis. *J. Lab. Clin. Med.* (in press).

NUIJENS, J.H., HUIKBREGTS, C.C.M., COHEN, M. & 5 others (1987). Detection of activation of the contact system of coagulation *in vitro* and *in vivo*: quantitation of activated Hageman factor-C1-inhibitor and Kallikrein-C1-inhibitor complexes by specific radioimmunoassays. *Thromb. Haemost.*, **58**, 778.

NUIJENS, J.H., HUIJBREGTS, C.C.M., EERENBERG-BELMER, A.J.M. & 5 others (1988). Quantification of plasma factor XIIa-C1 inhibitor and kallikrein-C1 inhibitor complexes in sepsis. *Blood*, **72**, 1841.

OGNIBENE, F.P., ROSENBERG, S.A. LOTZE, M.T. & 4 others (1988). Interleukin-2 administration causes reversible hemodynamic changes and left ventricular dysfunction similar to those seen in septic shock. *Chest*, **94**, 750.

OLIVER, R.T.O. (1988). The clinical potential of Interleukin-2. *Br. J. Cancer*, **58**, 405.

OPPENHEIM, J.J. (1983). Interleukin-2 mediated immune interferon (IFN-γ) production by human T cells and T cell subsets. *J. Immunol.*, **130**, 1784.

PARKER, M.M. & PARRILLO, J.E. (1983). Septic shock. Hemodynamics and pathogenesis. *JAMA*, **250**, 3324.

PEREZ, H.D., OHTANI, O., BANDA, D., ONG, R., FUKUYAMA, K. & GOLDSTEIN, I.M. (1983). Generation of biologically active complement (C5) derived peptides by cathepsin H. *J. Immunol.*, **131**, 397.

ROSENBERG, S.A., LOTZE, M.J., YANG, J.C. & 4 others (1989). Experience with the use of high dose Interleukin-2 in the treatment of 652 cancer patients. *Ann. Surg.*, **61**, 474.

SLOTMAN, G.J., BURCHARD, K.W., WILLIAMS, J.J., D'AZEZZO, A. & YELLIN, S.A. (1986). Interaction of prostaglandins, activated complement and granulocytes in clinical sepsis and hypotension. *Surgery*, **90**, 744.

SLOW, W.K., THONG, G.K., MCCORMACK, J.G. & FERRANTE, A. (1988). Lymphocyte-neutrophil interactions: opposite effects of Interleukin-2 and tumor necrosis factor-beta on human neutrophil adherence. *Int. Arch. Allergy Appl. Immunol.*, **85**, 63.

STEVENS, J.H., O'HANLEY, P., SHAPIRO, J.M. & 5 others (1986). Effects of anti-C5a antibodies on the adult respiratory distress syndrome in septic primates. *J. Clin. Invest.*, **177**, 1812.

THIJS, L.G., HACK, C.E., STRACK VAN SCHIJNDEL, R.J.M. & 5 others (1990). Activation of the complement system during immunotherapy with Interleukin-2. Relation to the development of side effects. *J. Immunol.*, **144**, 2419.

TONNESEN, M.G., SMEDLY, L.A. & HENSON, P.M. (1984). Neutrophil endothelial cell interactions. Modulation of neutrophil adhesiveness induced by complement fragments C3a and C5a des. arg. and formyl-methionyl-leucyl-phenylalanine *in vitro*. *J. Clin. Invest.*, **74**, 1581.

WARD, P.A. & MARKS, R.M. (1989). The acute inflammatory reaction. *Curr. Opinion Immunol.*, **2**, 5.

WEST, W.H. (1989). Continuous infusion of recombinant Interleukin-2 (rIL-2) in adoptive cellular therapy of renal cell carcinoma and other malignancies. *Cancer Treat. Rev.*, **16** (Suppl A): 83.